# Systematic review of the incidence, presentation and management of gastroduodenal artery pseudoaneurysm after pancreatic resection

**DOI:** 10.1002/bjs5.50210

**Published:** 2019-09-30

**Authors:** B. Brodie, H. M. Kocher

**Affiliations:** ^1^ Barts and the London School of Medicine and Dentistry London UK; ^2^ Centre for Tumour Biology, Barts Cancer Institute Queen Mary University of London London UK; ^3^ Barts and the London Hepato‐Pancreato‐Biliary Centre The Royal London Hospital, Barts Health NHS Trust, Whitechapel London UK

## Abstract

**Background:**

Gastroduodenal artery (GDA) pseudoaneurysm is a serious complication following pancreatic resection, associated with high morbidity and mortality rates. This review aimed to report the incidence of GDA pseudoaneurysm after pancreatic surgery, and describe clinical presentation and management.

**Methods:**

MEDLINE and Embase were searched systematically for clinical studies evaluating postoperative GDA pseudoaneurysm. Incidence was calculated by dividing total number of GDA pseudoaneurysms by the total number of pancreatic operations. Additional qualitative data related to GDA pseudoaneurysm presentation and management following pancreatic resection were extracted and reviewed from individual reports.

**Results:**

Nine studies were selected for systematic review involving 4227 pancreatic operations with 55 GDA pseudoaneurysms, with a reported incidence of 1·3 (range 0·2–8·3) per cent. Additional data were extracted from 39 individual examples of GDA pseudoaneurysm from 14 studies. The median time for haemorrhage after surgery was at 15 (range 4–210) days. A preceding complication in the postoperative period was documented in four of 21 patients (67 per cent), and sentinel bleeding was observed in 14 of 20 patients (70 per cent). Postoperative complications after pseudoaneurysm management occurred in two‐thirds of the patients (14 of 21). The overall survival rate was 85 per cent (33 of 39).

**Conclusion:**

GDA pseudoaneurysm is a rare yet serious cause of haemorrhage after pancreatic surgery, with high mortality. The majority of the patients had a preceding complication. Sentinel bleeding was an important clinical indicator.

## Graphical abstract

The management of gastroduodenal artery (GDA) pseudoaneurysm is primarily endovascular, with the exact method dependent on vessel anatomy, clinical state of the patient, collateral hepatic blood flow and interval from index surgery.

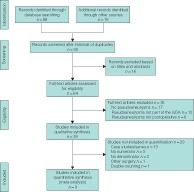

Remains an unusual but important complication

## Abstract (es)

**Antecedentes:**

El pseudoaneurisma (PA) de la arteria gastroduodenal (*gastroduodenal artery*, GDA) es una complicación grave después de la resección pancreática que conlleva elevadas tasas altas de morbilidad y mortalidad. Esta revisión tiene como objetivo estudiar la incidencia de PA de la GDA tras cirugía pancreática y describir la forma de presentación clínica y el tratamiento.

**Métodos:**

Se realizó una búsqueda sistemática en MEDLINE y EMBASE de los estudios clínicos que analizasen el PA postoperatorio de la GDA. Se calculó la incidencia dividiendo el número total de PA de GDA por el número total de intervenciones pancreáticas. De los informes de cada caso, se extrajeron los datos cualitativos relacionados con la forma de presentación y el tratamiento del PA de la GDA tras la resección pancreática.

**Resultados:**

Para la revisión sistemática se seleccionaron nueve estudios con 4.227 intervenciones sobre el páncreas y 55 PA de la GDA (incidencia 1,30% (rango 0,22‐8,33%). Se obtuvieron, además, datos individuales de 39 casos de PA de la GDA en 14 estudios. La hemorragia se presentó, como mediana, el día 15 (rango: 4‐210) del postoperatorio. Fue precedida de una complicación postoperatoria en el 66,7% de los casos y se observó una hemorragia centinela en el 70,0% de los pacientes. En dos tercios de los pacientes hubo complicaciones postoperatorias después del tratamiento del PA y la supervivencia global fue del 84,6%.

**Conclusión:**

Los PA de la GDA son una causa poco frecuente, pero grave, de hemorragia después de la cirugía pancreática, con una elevada mortalidad. La mayoría de los pacientes presentaron alguna complicación previa. La hemorragia centinela fue un indicador clínico de importancia.

## Introduction

Mortality from pancreatic resection has fallen significantly over the past few decades, especially in experienced centres^1^, ^2^, ^3^. Morbidity, including delayed gastric emptying, anastomotic leak and pancreatic fistula, remains high, affecting around 20–40 per cent of patients^4^, ^5^, ^6^. Postoperative haemorrhage is less common, but is a life‐threatening event with an estimated mortality rate of 20–50 per cent^7^, ^8^. Early‐onset haemorrhage is rare, and generally occurs within 24 h, usually due to technical failures. Delayed haemorrhage occurring days or weeks after surgery occurs for a variety of reasons, but one cause of massive haemorrhage is from the formation of visceral arterial pseudoaneurysms. Although several arteries have been shown to be vulnerable to pseudoaneurysm formation, observational studies indicate that pseudoaneurysms of the gastroduodenal artery (GDA) are the most common^9^, ^10^, ^11^.

The aim of this systematic review was to synthesize existing evidence regarding the incidence, clinical presentation and management of GDA pseudoaneurysms after pancreatic surgery.

## Methods

Studies evaluating GDA pseudoaneurysm formation after pancreatic surgery were identified by means of database searches of MEDLINE and Embase. In Embase the terms used were: ‘false‐aneurysm’ AND ‘gastroduodenal artery’ AND ‘pancreaticoduodenectomy’ OR ‘pancreatectomy’ OR ‘distal pancreatectomy’ OR ‘pylorus preserving pancreaticoduodenectomy’ OR ‘pancreas surgery’. In MEDLINE the terms used were: ‘false aneurysm’ OR ‘pseudoaneurysm’ AND ‘gastroduodenal artery’ AND ‘pancreaticoduodenectomy’ OR ‘pancreas surgery’ OR ‘pancreatectomy’ OR ‘distal pancreatectomy’ OR ‘pylorus preserving pancreaticoduodenectomy’.

A manual reference search was also performed to identify additional observational studies. No language restrictions were applied. Inclusion criteria were: manuscript published in a peer‐reviewed journal until 2017, investigating adult patients aged over 18 years, undergoing pancreatic surgery for any indication, developing GDA pseudoaneurysm, and reporting clinical outcomes of interest. The authors independently reviewed all relevant titles and abstracts, and all disagreements were resolved by consensus. Observational studies that reported both the number of GDA pseudoaneurysms and the total number of pancreatic operations performed were used for quantitative analysis of incidence. Qualitative information also relevant to the clinical presentation and management of GDA pseudoaneurysms was extracted from individual cases and collated. Data extracted included index surgery, sentinel bleeding defined as haemorrhage that occurred in the gastrointestinal tract (intraluminal) or intra‐abdominally (through a surgical drain) between 6 h and 10 days before a massive haemorrhage in the postoperative setting, day of postoperative bleeding, diagnostic method, management, other postoperative complications and mortality. GDA pseudoaneurysms were confirmed either radiologically or during surgery in all studies.

## Results

**Figure 1:**
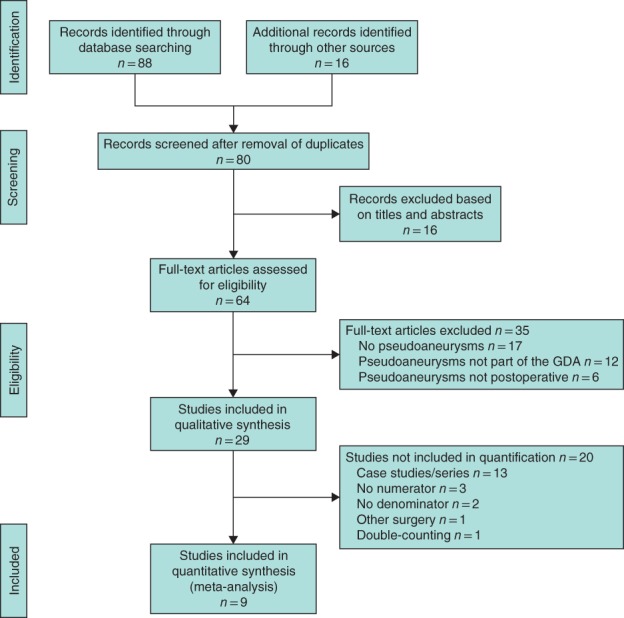
PRISMA diagram for the review GDA, gastroduodenal artery.

A PRISMA flow diagram is shown in *Fig*. 1. Some 88 studies were initially identified, 80 were screened, and 29 fulfilled the inclusion criteria. Of these, 13 studies^12^, ^13^, ^14^, ^15^, ^16^, ^17^, ^18^, ^19^, ^20^, ^21^, ^22^, ^23^, ^24^ were not included in the quantification as they were case series dealing exclusively with GDA pseudoaneurysms.

Five further studies were excluded as they did not provide the rate of GDA pseudoaneurysm in the postoperative period^25^, ^26^, ^27^, or the total number of pancreatic operations performed^28^, ^29^. One study^30^ was excluded because it also included procedures not involving the pancreas (hepatic resection and gastrojejunostomy). Finally, one study^9^ was removed as it recruited patients from the same institution as another report^31^, but over a shorter period.

All of the nine manuscripts^10^, ^11^, ^31^, ^32^, ^33^, ^34^, ^35^, ^36^, ^37^ selected for quantitative analysis were single‐centre observational studies (*Table* 1), mostly reporting on GDA pseudoaneurysms following pancreatoduodenectomy. One study^33^ included both pancreatoduodenectomy and modified pylorus‐preserving pancreatoduodenectomy, while another^10^ included several pancreatic procedures. Data from 39 patients with GDA pseudoaneurysms were extracted from 14 studies for systematic review of clinical presentation and management (*Table* 2).

**Table 1:** Studies included for determination of incidence

Reference	Country	Study interval	Surgical procedures	Total no. of operations	Total no. of GDA pseudoaneurysms	Incidence (%)
Adam *et al*.^11^	Turkey	January 1995 to January 2013	PD	342	7	2·0
Suzuki *et al*.^33^	Japan	January 2012 to July 2016	PD	88	5	6
Jeong *et al*.^31^	South Korea	October 1994 to December 2012	PD	1905	18	0·9
Yada *et al*.^32^	Japan	1982–2010	PD + PPPD	361	1	0·3
Loveček *et al*.^34^	Czech Republic	2006–2015	PD	449	1	0·2
Fujii *et al*.^10^	Japan	January 1993 to December 2005	PD + PPPD + DP + SR + HPD + TP	351	3	0·9
Rajarathinam *et al*.^35^	India	January 1998 to December 2007	PD	458	2	0·4
Hur *et al*.^36^	South Korea	March 2003 to March 2008	PD	192	16	8·3
Sato *et al*.^37^	Japan	January 1992 to December 1997	PD	81	2	2
Total				4227	55	1·3

GDA, gastroduodenal artery; PD, pancreatoduodenectomy; PPPD, pylorus‐preserving pancreatoduodenectomy; DP, distal pancreatectomy; SR, segmental resection; HPD, pancreatoduodenectomy plus hepatic resection; TP, total pancreatectomy.

**Table 2:** Data extracted for 39 patients with gastroduodenal artery pseudoaneurysm

Reference	Patient no.	Age (years)	Sex	Surgery	Complication	Sentinel bleed	POD of bleed	Diagnostic method	Management	Survival
Adam *et al*.^11^	1	43	M	PD	None	Yes	45	Angiography	Selective embolization	Yes
	2	68	F	PD	Abscess	Yes	10	Angiography	Selective embolization	Yes
	3	59	M	PD	Abscess	Yes	4	Angiography	TAE of CHA	Yes
	4	41	M	PD	Abscess	Yes	23	Angiography	TAE of CHA	Yes
	5	63	M	PD	Abscess	Yes	14	Angiography	Selective embolization	No
	6	72	F	PD	Abscess	Yes	25	Angiography	Selective embolization	Yes
	7	51	M	PD	None	Yes	7	Angiography	TAE of CHA	Yes
Fujii *et al*.^10^	1	–	–	HPD	Pancreatic leak	–	10	Angiography	TAE of CHA	Yes
	2	–	–	PD	Pancreatic fistula	–	11	Angiography	Relaparotomy	Yes
	3	–	–	HPD	Pancreatic leak	–	7	Angiography	Relaparotomy	No
Rajarathinam *et al*.^35^	1	52	M	PD	Pancreatic fistula	No	17	Angiography	Relaparotomy	Yes
	2	67	M	PD	Intra‐abdominal abscess	Yes	17	Angiography	Relaparotomy	No
Hur *et al*.^36^	1	–	–	PPPD	–	–	8	–	TAE of CHA	No
	2	–	–	PPPD	–	–	6	–	TAE of CHA	Yes
	3	–	–	PD	–	–	23	–	Selective embolization	Yes
	4	–	–	LPD	–	–	15	–	TAE of CHA	Yes
	5	–	–	LPD	–	–	7	–	Selective embolization	Yes
	6	–	–	PPPD	–	–	12	–	TAE of CHA	Yes
	7	–	–	PPPD	–	–	11	–	Selective embolization	No
	8	–	–	PPPD	–	–	7	–	TAE of CHA	Yes
	9	–	–	PPPD	–	–	8	–	TAE of CHA	Yes
	10	–	–	PPPD	–	–	19	–	TAE of CHA	Yes
	11	–	–	PPPD	–	–	7	–	TAE of CHA	Yes
	12	–	–	PPPD	–	–	19	–	TAE of CHA	Yes
	13	–	–	PPPD	–	–	8	–	TAE of CHA	Yes
	14	–	–	PPPD	–	–	14	–	TAE of CHA	Yes
	15	–	–	HPD	–	–	9	–	TAE of CHA	Yes
	16	–	–	PPPD	–	–	13	–	TAE of CHA	Yes
Miyazawa *et al*.^13^	1	71	M	PD	Postoperative bleed	No	180	Contrast CT	Stenting	Yes
Loveček *et al*.^14^	1	58	M	PD	None	Yes	18	Angiography	Stenting	Yes
Mazza *et al*.^16^	1	61	M	MSR	None	No	210	Contrast CT	Selective embolization	Yes
Huang *et al*.^17^	1	72	M	PD	–	Yes	17	–	Selective embolization	Yes
	2	65	F	Duodenum‐preserving pancreatic resection	–	Yes	30	–	Selective embolization	No
Noun *et al*.^18^	1	58	M	PD	Pancreatic fistula	Yes	19	Angiography	Selective embolization	Yes
Orsenigo *et al*.^21^	1	38	M	SPK	AV fistula	No	15	MR angiography	Selective embolization	Yes
Sugimoto *et al*.^22^	1	62	M	PD	None	No	120	Angiography	TAE of CHA	Yes
Born *et al*.^23^	1	42	M	Lateral pancreatojejunostomy	None	Yes	21	Angiography	TAE of CHA	Yes
Teramoto *et al*.^24^	1	70	M	PD	Pancreatic leak	No	34	Angiography	Selective embolization	Yes
Okuno *et al*.^30^	1	46	F	PD	None	Yes	62	Angiography	Selective embolization	Yes
Overall	39	60*	16 M, 4 F		14 of 21	14 of 20	15 (4–210)*			33 of 39

* Median (range) value. POD, postoperative day; PD, pancreatoduodenectomy; TAE, transarterial embolization; CHA, common hepatic artery; HPD, pancreatectomy plus hepatic resection; PPPD, pylorus‐preserving pancreatoduodenectomy; LPD, laparoscopic pancreatoduodenectomy; MSR, middle segment resection; SPK, simultaneous pancreas–kidney transplant.

A total of 55 GDA pseudoaneurysms were identified in the postoperative period following 4227 pancreatic procedures, with a reported incidence of 1·3 (range 0·2–8·3) per cent (*Table* 1). Most patients who developed GDA pseudoaneurysm had a preceding complication in the postoperative period (14 of 21), including abscesses (6 patients), pancreatic fistulas (3) and pancreatic leaks (3). Three studies^10^, ^22^, ^38^ reported the formation of pseudoaneurysms away from the cut edge of the pancreas and in the absence of pancreatic fistulas. Sentinel bleeding was reported in 14 of 20 patients (70 per cent). The median time for postoperative haemorrhage was at 15 (range 4–210) days.

Diagnostic procedures were reported for 21 patients; 18 GDA pseudoaneurysms were detected by angiography.

Thirty‐five of the 39 patients (90 per cent) were treated using an endovascular approach. Nineteen (49 per cent) were managed using transarterial embolization (TAE) of the GDA via the common hepatic artery (CHA), and 14 (36 per cent) by selective embolization of the pseudoaneurysm. Stenting was employed in two patients (5 per cent), and only four (10 per cent) were treated by emergency laparotomy. The overall survival rate was 85 per cent (33 of 39).

## Discussion

The GDA is the most common site for pseudoaneurysm formation after pancreatic surgery^9^, ^10^, ^11^, and its rupture in the postoperative period has long been recognized as a cause of substantial morbidity and mortality^7^, ^8^. GDA pseudoaneurysms are rare. The present analysis suggests that they occur in 0·2–8·3 per cent of pancreatic resections. It should be noted, however, that studies included in this review were all high‐volume resectional centres.

In this series, two‐thirds of the patients (4 of 21) had a preceding complication following pancreatic resection. Most authors favoured the hypothesis that lytic, enzyme‐rich, pancreatic fluid from a pancreatic anastomotic leak could result in autodigestion of GDA vessel wall owing to its proximity to the pancreatic anastomosis.

Interestingly, a few studies^10^, ^22^, ^38^ reported the formation of pseudoaneurysms at distance from the pancreatic anastomosis and in the absence of an overt pancreatic fistula, suggesting that minor iatrogenic injury, such as skeletonization of the vessel wall during extensive lymphadenectomy, may lead to vessel weakening and subsequent pseudoaneurysm formation.

Various techniques have been suggested to reduce the chance of pseudoaneurysm formation, including the ‘wrapping’ technique^25^, ^39^, ^40^, ^41^, ^42^, ^43^, ^44^, ^45^, in which the exposed retroperitoneal vessels are covered with omentum or the falciform ligament. Others^46^ have suggested leaving 1 cm at the origin of the GDA stump to minimize the likelihood of lytic pancreatic juices coming into contact with the vessel.

Recognition of a sentinel bleed may help in early management, this being a feature in most patients^9^, ^37^, ^47^. Although sentinel bleeding was associated with poor outcome in some series^6^, ^35^, few authors discussed the importance of immediate angiography after a sentinel bleed to look for the possibility of a ruptured pseudoaneurysm^6^, ^37^. Although angiography also has the added benefit of allowing transition to endovascular treatment, a number of reports noted that negative findings cannot be used to exclude a bleeding pseudoaneurysm^26^, as bleeding can be intermittent or the rate of bleeding is below the detection limit of the equipment^7^, ^9^, ^37^, ^48^, ^49^.

Surgical intervention has been largely replaced by interventional radiology^50^, ^51^, ^52^, ^53^, ^54^. Some older studies^26^, ^55^ advocated surgery in the context of additional intra‐abdominal complications such as pancreatic fistula, but more recent series^11^, ^36^ have documented the superiority of endovascular management. A recent meta‐analysis^56^ of non‐randomized studies comparing endovascular management and laparotomy for delayed massive haemorrhage suggested lower complication and mortality rates in the endovascular group.

The endovascular management of pseudoaneurysms varied, reflecting the location and size of the pseudoaneurysm and probably institutional preferences for approach and embolization technique, and materials. TAE of the GDA was conducted via either the CHA or the superior mesenteric artery to achieve both proximal and distal occlusion, to exclude the pseudoaneurysm and prevent backflow from collateral circulation^57^. Such an approach should consider patency of the portal venous system^41^ as TAE distal and proximal to the GDA pseudoaneurysm can cause complete occlusion of the CHA, leading to liver infarction (reported range 30–66 per cent)^29^, ^37^, ^58^, as well as hepatic failure and abscess formation^58^. Covered stents represented the alternative to TAE. The key advantage over TAE would be in maintaining patency of the CHA and reducing the risk of hepatic infarction, although accurate stent deployment might be technically more challenging and time‐consuming than TAE^36^, ^57^. Despite these issues, stenting seems to be preferred in more recent series, on the grounds that selective embolization of the GDA stump or pseudoaneurysm seems to be associated with high rates of recurrent bleeding^36^, ^42^.

Limitations of this study include heterogeneity of the included studies, the descriptors used and study sizes. The absence of any prospective registers or clinical trials on this topic needs to be addressed.

## Disclosure

The authors declare no conflict of interest.

## References

[1] Saeger HD , Schwall G , Trede M . Standard Whipple in chronic pancreatitis In Standards in Pancreatic Surgery, Beger HG, Büchler M, Malfertheiner P (eds). Springer: Berlin, 1993; 385–391.

[2] Cameron JL , Pitt HA , Yeo CJ , Lillemoe KD , Kaufman HS , Coleman J . One hundred and forty‐five consecutive pancreaticoduodenectomies without mortality. Ann Surg 1993; 217: 430–435. 8098202 10.1097/00000658-199305010-00002 PMC1242815

[3] Büchler MW , Friess H , Müller MW , Wheatley AM , Beger HG . Randomized trial of duodenum‐preserving pancreatic head resection *versus* pylorus‐preserving Whipple in chronic pancreatitis. Am J Surg 1995; 169: 65–69. 7818000 10.1016/s0002-9610(99)80111-1

[4] Schlitt HJ , Schmidt U , Simunec D , Jäger M , Aselmann H , Neipp M *et al* Morbidity and mortality associated with pancreatogastrostomy and pancreatojejunostomy following partial pancreatoduodenectomy. Br J Surg 2002; 89: 1245–1251. 12296891 10.1046/j.1365-2168.2002.02202.x

[5] Yeo CJ , Cameron JL , Sohn TA , Lillemoe KD , Pitt HA , Talamini MA *et al* Six hundred fifty consecutive pancreaticoduodenectomies in the 1990s: pathology, complications, and outcomes. Ann Surg 1997; 226: 248–257. 9339931 10.1097/00000658-199709000-00004 PMC1191017

[6] Yekebas EF , Wolfram L , Cataldegirmen G , Habermann CR , Bogoevski D , Koenig AM *et al* Postpancreatectomy hemorrhage: diagnosis and treatment: an analysis in 1669 consecutive pancreatic resections. Ann Surg 2007; 246: 269–280. 17667506 10.1097/01.sla.0000262953.77735.db PMC1933568

[7] Brodsky JT , Turnbull AD . Arterial hemorrhage after pancreatoduodenectomy. The ‘sentinel bleed’. Arch Surg 1991; 126: 1037–1040. 1863209 10.1001/archsurg.1991.01410320127019

[8] Lerut JP , Gianello PR , Otte JB , Kestens PJ . Pancreaticoduodenal resection. Surgical experience and evaluation of risk factors in 103 patients. Ann Surg 1984; 199: 432–437. 6712319 10.1097/00000658-198404000-00010 PMC1353362

[9] Lee HG , Heo JS , Choi SH , Choi DW . Management of bleeding from pseudoaneurysms following pancreaticoduodenectomy. World J Gastroenterol 2010; 16: 1239–1244. 20222168 10.3748/wjg.v16.i10.1239 PMC2839177

[10] Fujii Y , Shimada H , Endo I , Yoshida K , Matsuo K , Takeda K *et al* Management of massive arterial hemorrhage after pancreatobiliary surgery: does embolotherapy contribute to successful outcome? J Gastrointest Surg 2007; 11: 432–438. 17436126 10.1007/s11605-006-0076-9 PMC1852380

[11] Adam G , Tas S , Cinar C , Bozkaya H , Adam F , Uysal F *et al* Endovascular treatment of delayed hemorrhage developing after the pancreaticoduodenectomy procedure. Wien Klin Wochenschr 2014; 126: 416–421. 24865770 10.1007/s00508-014-0557-x

[12] Budzyński J , Meder G , Suppan K . Giant gastroduodenal artery pseudoaneurysm as a pancreatic tumor and cause of acute bleeding into the digestive tract. Prz Gastroenterol 2016; 11: 299–301. 28053687 10.5114/pg.2016.61478 PMC5209468

[13] Miyazawa R , Kamo M , Nishiyama T , Ohigashi S , Yagihashi K . Covered stent placement using ‘pull‐through’ technique for a gastroduodenal artery stump pseudoaneurysm after pancreaticoduodenectomy. J Vasc Interv Radiol 2016; 27: 1743–1745. 27926407 10.1016/j.jvir.2016.06.033

[14] Loveček M , Havlík R , Köcher M , Vomáčková K , Neoral C . Pseudoaneurysm of the gastroduodenal artery following pancreatoduodenectomy. Stenting for hemorrhage. Wideochir Inne Tech Maloinwazyjne 2014; 9: 297–301. 25097705 10.5114/wiitm.2011.38178 PMC4105658

[15] Nakatsuka H , Sawatsubashi T , Morioka N , Shimizu T , Kanda T . [Use of the round ligament of the liver to prevent postpancreatectomy haemorrhage.] Gan To Kagaku Ryoho 2013; 40: 1903–1905. 24393960

[16] Mazza E , Abdulcadir D , Raspanti C , Acquafresca M . A challenging case of epigastric pain: diagnosis and mini‐invasive treatment of a large gastroduodenal artery pseudoaneurysm. BMJ Case Rep 2012; 2012: bcr0220125873. 10.1136/bcr.02.2012.5873 PMC3448759 22983999

[17] Huang YK , Lu MS , Tsai FC , Ko PJ , Hsieh HC , Lin PJ . A forgotten complication following pancreatic resection. Visceral artery pseudo‐aneurysms. Saudi Med J 2007; 28: 973–975. 17530126

[18] Noun R , Zeidan S , Tohme‐Noun C , Smayra T , Sayegh R . Biliary ischemia following embolization of a pseudoaneurysm after pancreaticoduodenectomy. JOP 2006; 7: 427–431. 16832142

[19] Balducci G , Dente M , Ferri M , Rebonato A , La Torre M , Mercantini P . [Bleeding caused by pseudoaneurysm rupture after pancreaticoduodenectomy.] G Chir 2006; 27: 318–320. 17064491

[20] Santoro R , Carlini M , Carboni F , Nicolas C , Santoro E . Delayed massive arterial hemorrhage after pancreaticoduodenectomy for cancer. Management of a life‐threatening complication. Hepatogastroenterology 2003; 50: 2199–2204. 14696498

[21] Orsenigo E , De Cobelli F , Salvioni M , Cristallo M , Fiorina P , Del Maschio A *et al* Successful endovascular treatment for gastroduodenal artery pseudoaneurysm with an arteriovenous fistula after pancreas transplantation. Transpl Int 2003; 16: 694–696. 12819860 10.1007/s00147-003-0611-5

[22] Sugimoto H , Kaneko T , Ishiguchi T , Takai K , Ohta T , Yagi Y *et al* Delayed rupture of a pseudoaneurysm following pancreatoduodenectomy: report of a case. Surg Today 2001; 31: 932–935. 11759894 10.1007/s005950170039

[23] Born LJ , Madura JA , Lehman GA . Endoscopic diagnosis of a pancreatic pseudoaneurysm after lateral pancreaticojejunostomy. Gastrointest Endosc 1999; 49: 382–384. 10049425 10.1016/s0016-5107(99)70018-0

[24] Teramoto K , Kawamura T , Takamatsu S , Noguchi N , Arii S . A case of hepatic artery embolization and partial arterialization of the portal vein for intraperitoneal, hemorrhage after a pancreaticoduodenectomy. Hepatogastroenterology 2003; 50: 1217–1219. 14571702

[25] Ray S , Sanyal S , Ghatak S , Sonar PK , Das S , Khamrui S *et al* Falciform ligament flap for the protection of the gastroduodenal artery stump after pancreaticoduodenectomy: a single center experience. J Visc Surg 2016; 153: 9–13. 26526210 10.1016/j.jviscsurg.2015.10.007

[26] de Castro SM , Kuhlmann KF , Busch OR , van Delden OM , Laméris JS , van Gulik TM *et al* Delayed massive hemorrhage after pancreatic and biliary surgery: embolization or surgery? Ann Surg 2005; 241: 85–91. 15621995 10.1097/01.sla.0000150169.22834.13 PMC1356850

[27] Magge D , Zenati M , Lutfi W , Hamad A , Zureikat AH , Zeh HJ *et al* Robotic pancreatoduodenectomy at an experienced institution is not associated with an increased risk of post‐pancreatic hemorrhage. HPB (Oxford) 2018; 20: 448–455. 29366816 10.1016/j.hpb.2017.11.005

[28] Kalva SP , Yeddula K , Wicky S , Fernandez del Castillo C , Warshaw AL . Angiographic intervention in patients with a suspected visceral artery pseudoaneurysm complicating pancreatitis and pancreatic surgery. Arch Surg 2011; 146: 647–652. 21339414 10.1001/archsurg.2011.11

[29] Gwon DI , Ko GY , Sung KB , Shin JH , Kim JH , Yoon HK . Endovascular management of extrahepatic artery hemorrhage after pancreatobiliary surgery: clinical features and outcomes of transcatheter arterial embolization and stent‐graft placement. AJR Am J Roentgenol 2011; 196: W627–W634. 21512055 10.2214/AJR.10.5148

[30] Okuno A , Miyazaki M , Ito H , Ambiru S , Yoshidome H , Shimizu H *et al* Nonsurgical management of ruptured pseudoaneurysm in patients with hepatobiliary pancreatic diseases. Am J Gastroenterol 2001; 96: 1067–1071. 11316148 10.1111/j.1572-0241.2001.03691.x

[31] Jeong J , Choi SH , Choi DW , Heo JS , Kim DH , Lee H . Management of delayed arterial hemorrhage following pancreaticoduodenectomy: a single‐center experience. HPB (Oxford) 2014; 16: 133.

[32] Yada K , Kawano Y , Komori Y , Masuda T , Hirashita T , Eguchi H *et al* Perioperative outcomes of pancreatoduodenectomy in elderly patients. J Gastroenterol Hepatol 2011; 26: 233.

[33] Suzuki K , Mori Y , Komada T , Matsushima M , Ota T , Naganawa S . Stent‐graft treatment for bleeding superior mesenteric artery pseudoaneurysm after pancreaticoduodenectomy. Cardiovasc Intervent Radiol 2009; 32: 762–766. 19184196 10.1007/s00270-009-9502-1

[34] Loveček M , Skalický P , Köcher M , Černá M , Prášil V , Holusková I *et al* [Postpancreatectomy haemorrhage (PPH), prevalence, diagnosis and management.] Rozhl Chir 2016; 95: 350–357. 27653303

[35] Rajarathinam G , Kannan DG , Vimalraj V , Amudhan A , Rajendran S , Jyotibasu D *et al* Post pancreaticoduodenectomy haemorrhage: outcome prediction based on new ISGPS Clinical severity grading. HPB (Oxford) 2008; 10: 363–370. 18982153 10.1080/13651820802247086 PMC2575673

[36] Hur S , Yoon CJ , Kang SG , Dixon R , Han HS , Yoon YS *et al* Transcatheter arterial embolization of gastroduodenal artery stump pseudoaneurysms after pancreaticoduodenectomy: safety and efficacy of two embolization techniques. J Vasc Interv Radiol 2011; 22: 294–301. 21353982 10.1016/j.jvir.2010.11.020

[37] Sato N , Yamaguchi K , Shimizu S , Morisaki T , Yokohata K , Chijiiwa K *et al* Coil embolization of bleeding visceral pseudoaneurysms following pancreatectomy: the importance of early angiography. Arch Surg 1998; 133: 1099–1102. 9790208 10.1001/archsurg.133.10.1099

[38] Reber PU , Baer HU , Patel AG , Wildi S , Triller J , Büchler MW . Superselective microcoil embolization: treatment of choice in high‐risk patients with extrahepatic pseudoaneurysms of the hepatic arteries. J Am Coll Surg 1998; 186: 325–330. 9510264 10.1016/s1072-7515(98)00032-5

[39] Sato A , Yamada T , Takase K , Matsuhashi T , Higano S , Kaneda T *et al* The fatal risk in hepatic artery embolization for hemostasis after pancreatic and hepatic surgery: importance of collateral arterial pathways. J Vasc Interv Radiol 2011; 22: 287–293. 21353981 10.1016/j.jvir.2010.11.023

[40] Gaudon C , Soussan J , Louis G , Moutardier V , Gregoire E , Vidal V . Late postpancreatectomy hemorrhage: predictive factors of morbidity and mortality after percutaneous endovascular treatment. Diagn Interv Imaging 2016; 97: 1071–1077. 27592120 10.1016/j.diii.2016.08.003

[41] Tani M , Kawai M , Hirono S , Hatori T , Imaizumi T , Nakao A *et al* Use of omentum or falciform ligament does not decrease complications after pancreaticoduodenectomy: nationwide survey of the Japanese Society of Pancreatic Surgery. Surgery 2012; 151: 183–191. 21982073 10.1016/j.surg.2011.07.023

[42] Maeda A , Ebata T , Kanemoto H , Matsunaga K , Bando E , Yamaguchi S *et al* Omental flap in pancreaticoduodenectomy for protection of splanchnic vessels. World J Surg 2005; 29: 1122–1126. 16132400 10.1007/s00268-005-7900-3

[43] Ramia JM , de la Plaza R , Adel F , Ramiro C , Arteaga V , Garcia‐Parreño J . Wrapping in pancreatic surgery: a systematic review. ANZ J Surg 2014; 84: 921–924. 25720806 10.1111/ans.12491

[44] Choi SB , Lee JS , Kim WB , Song TJ , Suh SO , Choi SY . Efficacy of the omental roll‐up technique in pancreaticojejunostomy as a strategy to prevent pancreatic fistula after pancreaticoduodenectomy. Arch Surg 2012; 147: 145–150. 22351908 10.1001/archsurg.2011.865

[45] Matsuda H , Sadamori H , Umeda Y , Shinoura S , Yoshida R , Satoh D *et al* Preventive effect of omental flap in pancreaticoduodenectomy against postoperative pseudoaneurysm formation. Hepatogastroenterology 2012; 59: 578–583. 21940374 10.5754/hge11452

[46] Turrini O , Moutardier V , Guiramand J , Lelong B , Bories E , Sannini A *et al* Hemorrhage after duodenopancreatectomy: impact of neoadjuvant radiochemotherapy and experience with sentinel bleeding. World J Surg 2005; 29: 212–216. 15654661 10.1007/s00268-004-7557-3

[47] Koukoutsis I , Bellagamba R , Morris‐Stiff G , Wickremesekera S , Coldham C , Wigmore SJ *et al* Haemorrhage following pancreaticoduodenectomy: risk factors and the importance of sentinel bleed. Dig Surg 2006; 23: 224–228. 16874003 10.1159/000094754

[48] Shankar S , Russell RC . Haemorrhage in pancreatic disease. Br J Surg 1989; 76: 863–866. 2765846 10.1002/bjs.1800760833

[49] van Berge Henegouwen MI , Allema JH , van Gulik TM , Verbeek PC , Obertop H , Gouma DJ . Delayed massive haemorrhage after pancreatic and biliary surgery. Br J Surg 1995; 82: 1527–1531. 8535810 10.1002/bjs.1800821124

[50] Bassi C , Falconi M , Salvia R , Mascetta G , Molinari E , Pederzoli P . Management of complications after pancreaticoduodenectomy in a high volume centre: results on 150 consecutive patients. Dig Surg 2001; 18: 453–457. 11799295 10.1159/000050193

[51] Robinson K , Rajebi MR , Zimmerman N , Zeinati C . Post‐pancreaticoduodenectomy hemorrhage of unusual origin: treatment with endovascular embolization and the value of preoperative CT angiography. J Radiol Case Rep 2013; 7: 29–36. 23705050 10.3941/jrcr.v7i4.1254 PMC3661432

[52] Johnson MA , Chidambaram S . Current management protocol in peripancreatic pseudoaneurysms. Pancreatology 2011; 11: 40.21464586

[53] Arata MA , Cope C . Principles used in the management of visceral aneurysms. Tech Vasc Interv Radiol 2000; 3: 124–129.

[54] Saad NE , Saad WE , Davies MG , Waldman DL , Fultz PJ , Rubens DJ . Pseudoaneurysms and the role of minimally invasive techniques in their management. Radiographics 2005; 25(Suppl 1): S173–S189. 16227490 10.1148/rg.25si055503

[55] Blanc T , Cortes A , Goere D , Sibert A , Pessaux P , Belghiti J *et al* Hemorrhage after pancreaticoduodenectomy: when is surgery still indicated? Am J Surg 2007; 194: 3–9. 17560900 10.1016/j.amjsurg.2006.08.088

[56] Limongelli P , Khorsandi SE , Pai M , Jackson JE , Tait P , Tierris J *et al* Management of delayed postoperative hemorrhage after pancreaticoduodenectomy: a meta‐analysis. Arch Surg 2008; 143: 1001–1007. 18936380 10.1001/archsurg.143.10.1001

[57] Ding X , Zhu J , Zhu M , Li C , Jian W , Jiang J *et al* Therapeutic management of hemorrhage from visceral artery pseudoaneurysms after pancreatic surgery. J Gastrointest Surg 2011; 15: 1417–1425. 21584822 10.1007/s11605-011-1561-3

[58] Otah E , Cushin BJ , Rozenblit GN , Neff R , Otah KE , Cooperman AM . Visceral artery pseudoaneurysms following pancreatoduodenectomy. Arch Surg 2002; 137: 55–59. 11772216 10.1001/archsurg.137.1.55

